# Lateral diffusion of CD14 and TLR2 in macrophage plasma membrane assessed by raster image correlation spectroscopy and single particle tracking

**DOI:** 10.1038/s41598-020-76272-2

**Published:** 2020-11-09

**Authors:** Sara Makaremi, Markus Rose, Suman Ranjit, Michelle A. Digman, Dawn M. E. Bowdish, Jose M. Moran-Mirabal

**Affiliations:** 1 School of Biomedical Engineering, McMaster University, Hamilton, ON Canada; 2 Department of Physics and Astronomy, McMaster University, Hamilton, ON Canada; 3 Department of Biochemistry, Molecular and Cellular Biology, Georgetown University, Washington, DC USA; 4 Department of Biomedical Engineering, University of California Irvine, Irvine, CA USA; 5 Department of Pathology and Molecular Medicine, McMaster University, Hamilton, ON Canada; 6 MG DeGroote Institute for Infectious Diseases, McMaster University, Hamilton, ON Canada; 7 Department of Chemistry and Chemical Biology, McMaster University, Hamilton, ON Canada

**Keywords:** Biophysics, Immunology

## Abstract

The diffusion of membrane receptors is central to many biological processes, such as signal transduction, molecule translocation, and ion transport, among others; consequently, several advanced fluorescence microscopy techniques have been developed to measure membrane receptor mobility within live cells. The membrane-anchored receptor cluster of differentiation 14 (CD14) and the transmembrane toll-like receptor 2 (TLR2) are important receptors in the plasma membrane of macrophages that activate the intracellular signaling cascade in response to pathogenic stimuli. The aim of the present work was to compare the diffusion coefficients of CD14 and TLR2 on the apical and basal membranes of macrophages using two fluorescence-based methods: raster image correlation spectroscopy (RICS) and single particle tracking (SPT). In the basal membrane, the diffusion coefficients obtained from SPT and RICS were found to be comparable and revealed significantly faster diffusion of CD14 compared with TLR2. In addition, RICS showed that the diffusion of both receptors was significantly faster in the apical membrane than in the basal membrane, suggesting diffusion hindrance by the adhesion of the cells to the substrate. This finding highlights the importance of selecting the appropriate membrane (i.e., basal or apical) and corresponding method when measuring receptor diffusion in live cells*.* Accurately knowing the diffusion coefficient of two macrophage receptors involved in the response to pathogen insults will facilitate the study of changes that occur in signaling in these cells as a result of aging and disease.

## Introduction

The plasma membrane is a highly fluid and dynamic environment, where lipids and proteins laterally diffuse within the lipid bilayer—a feature that enables key cellular processes involving the transport of biological species^1^. Several powerful fluorescence-based methods have been used to study the dynamics of membrane components, such as the diffusion and association of immunoreceptors^2–5^. These techniques are based on either ensemble measurements, where the diffusion coefficient is obtained from the average motion of groups of molecules, or single particle tracking where the mobility of individual particles is analyzed. Ensemble techniques include fluorescence recovery after photobleaching (FRAP), which bleaches a small area of the fluorescently labeled membrane with a brief intense illumination pulse. The diffusion coefficient is then determined by monitoring the fluorescence recovery that results as bleached molecules migrate out of the illuminated area and are replaced by fluorescent ones^6,7^. A second ensemble method is fluorescence correlation spectroscopy (FCS)^8,9^, which determines the diffusion by monitoring the fluctuations of the fluorescence signal in a fixed observation volume. FCS is used to measure fast (micro- to millisecond timescale) dynamics, binding and association kinetics, in a single fixed spot on the membrane^4^.

Raster image correlation spectroscopy (RICS)^10,11^ is in principle an extended version of FCS, with the addition of a spatial component using confocal laser-scanning microscopy (CLSM), which uses the raster scanning to capture the fluctuations in the intensity caused by the movement of fluorescent molecules. By measuring the intensity at one pixel for a very brief period of time followed by measuring the intensity of adjacent pixels immediately after, the intensities of pixels within each frame can be correlated pair-wise to identify characteristic decay times corresponding to dynamic processes, such as the diffusion of fluorescent particles through the detection volume^12^. The spatial correlation depends on the rate of diffusion, the pixel dwell time, and the size of pixels. Since RICS is typically implemented using CLSM, the reduction of out-of-focus signal enables measurements that are confined to a narrow plane in the cell. Additionally, the ability of the RICS analysis routine to separate slow and immobile fractions makes it possible to monitor the diffusion of heterogeneous particles. Thus, this technique is frequently used to measure the diffusion coefficient of proteins in live cells^13–16^.

Single particle tracking (SPT) is commonly used to measure the diffusion of membrane components in live cells^2,17,18^. SPT provides information on the trajectories of individual particles with spatial resolutions < 20 nm. SPT is typically performed on images acquired with a total internal reflection fluorescence (TIRF) microscope, where the acquisition of the intensities of all the pixels in each frame is done simultaneously and isolated particles are followed for relatively long periods of time (seconds to minutes), with capture frame rates of up to 40 kHz^2^. The images are then analyzed to precisely locate each fluorescent particle within the image and the trajectories are built by linking the positions of particles in consecutive frames.

The aim of the present study was to measure and compare the lateral diffusion of two key receptors involved in the recognition of pathogenic stimuli on the plasma membrane of macrophages, using two different fluorescence-based techniques for comparison: RICS and SPT. Transmembrane toll-like receptor 2 (TLR2) and glycosylphosphatidylinositol (GPI)-linked cluster of differentiation 14 (CD14) are both receptors expressed by macrophages, which are key in binding bacterial products and initiating inflammatory responses. TLRs are type I glycoproteins composed of extracellular, transmembrane, and intracellular signaling domains^19^. TLR2 binds the anchor motif of lipoproteins found on the surface of bacteria and responds to lipid-containing pathogen associated molecular patterns such as lipoteichoic acid (LTA) from Gram-positive bacteria^20^. CD14, on the other hand, is a pattern-recognition receptor that binds lipopolysaccharide (LPS) in Gram-negative and LTA in Gram-positive bacterial membranes^21^.

The spatial distribution of immunoreceptors and their lateral mobility in the plasma membrane impact receptor-mediated signaling^22^. Lateral clustering of receptors is essential for their activation since an increased local density of receptors enhances the efficiency of signal transduction whenever cooperation between multiple molecular players is required^22^. For example, TLR2 forms TLR-ligand complexes and initiates signaling through dimerization with other TLRs^19^. CD14 serves as a coreceptor of many TLRs, including TLR2^23^, and activates the intracellular signaling cascade and the innate immune response with the help of transmembrane receptors. Previous reports on the lateral diffusion of TLR2 and CD14 in other cell types have provided valuable insights into their dynamic behavior in the plasma membrane and their signaling mechanism. FRAP measurements on Chinese hamster ovary (CHO) cells transfected with TLR2 have revealed that upon stimulation these receptors become transiently confined within lipid rafts in the plasma membrane, which in turn promotes the formation of clusters that trigger signaling^24^. CD14 diffusion has also been studied in CHO using FRAP, which has shown a rapid transfer of bacterial component LPS from CD14 to other immobile receptor complexes such as heat shock proteins^25^. Similarly, it has been shown that CD14 binding to its ligands in human embryonic kidney cells enhances signaling through the TLR2 heterodimer complex by reducing the physical proximity of the ligand to the TLRs for more efficient delivery of the microbial component to the TLRs^26^, without binding to the complex^27^. Given the importance of TLR2 and CD14 in macrophage function, extracting the rates of diffusion of these and other related immunoreceptors can be invaluable in understanding their signaling mechanisms in response to bacterial products.

To our knowledge, this is the first study that measures the lateral diffusion of TLR2 and CD14 in the apical and basal plasma membranes of macrophages and directly compares the diffusion measurements with the aid of two fluorescence-based methods, SPT and RICS. Studying the dynamics of these receptors using two different techniques provides complementary insights into their diffusion behavior as well as a true comparison of the precision, advantages, and disadvantages of these techniques.

## Results

### Single particle tracking

**Figure 1:**
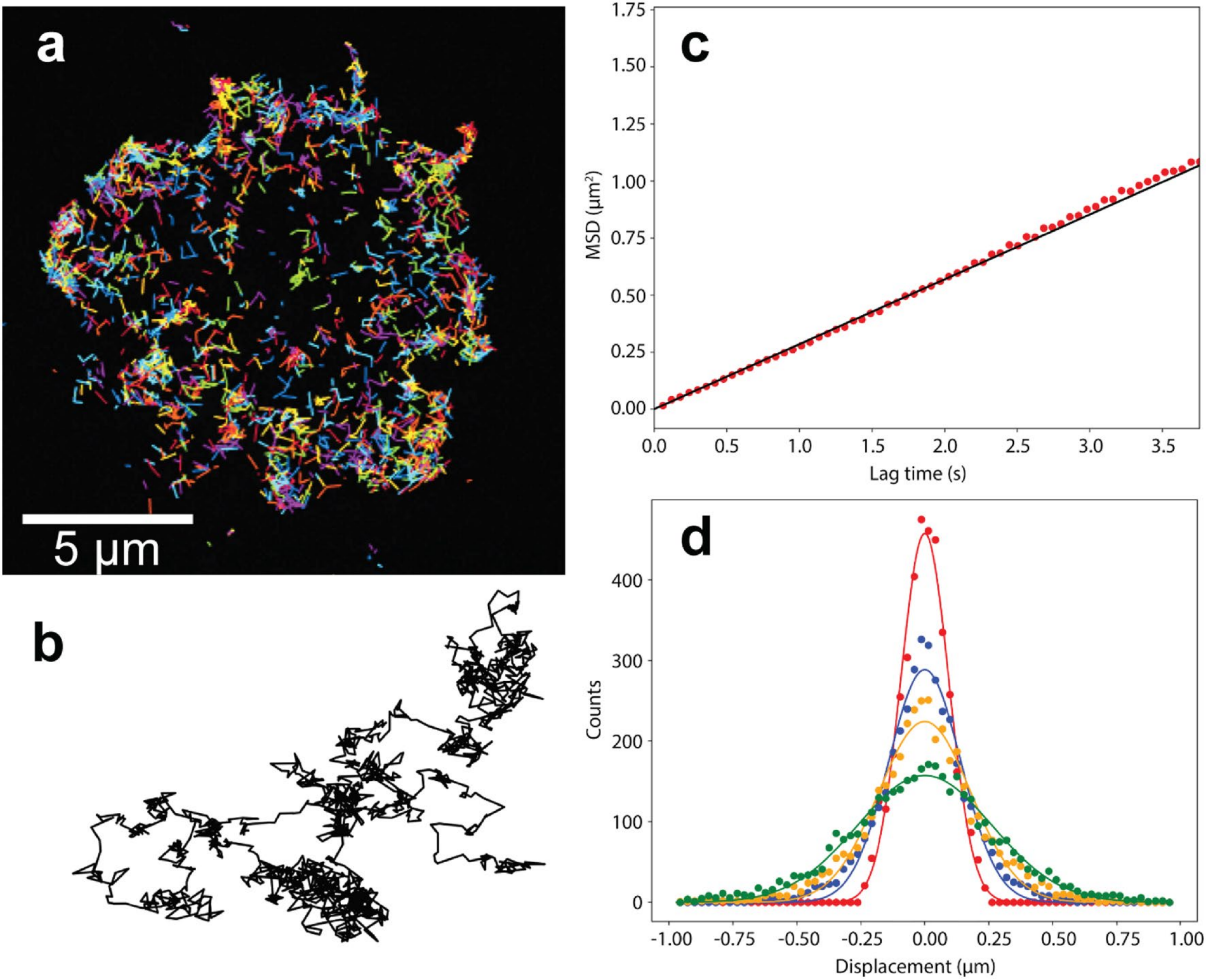
Single particle tracking analysis of diffusion in the plasma membrane of RAW 264.7 macrophages. (**a**) TIRF microscopy image of TLR2 on the basal membrane with tracks of individual particles generated from 300 frames (frame interval 60 ms). (**b**) Combined track generated from appending 134 individual trajectories. (**c**) Mean-squared displacement obtained from the combined track and the linear fit to equation < (Δ*r*)^2^ > (*τ*) = 4*Dτ* to determine diffusion coefficient. (**d**) Distribution of displacements for all trajectories at different lag times (red: *τ* = 0.06 s, blue: *τ* = 0.30 s, yellow: *τ* = 0.54 s, green: *τ* = 1.01 s). Normal distribution is fitted (solid lines) using Eq. (2).

**Figure 2:**
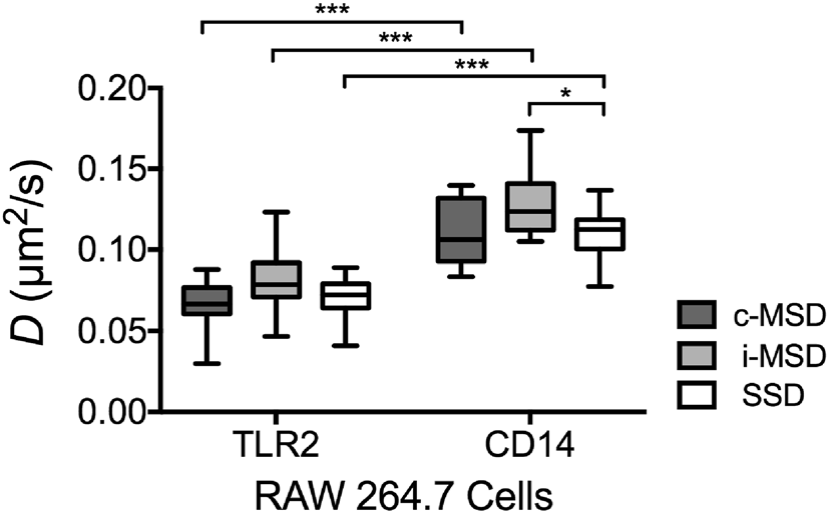
Diffusion measurements in the plasma membrane of RAW 264.7 macrophages obtained from single particle tracking and the mean-squared displacement analysis for combined (c-MSD) and individual (i-MSD) tracks and the step-size distribution (SSD). Data is from basal membranes of 15 cells for each type of receptor. Boxes show 25th–75th percentiles with whiskers extending to minimum and maximum values measured (*P ≤ 0.05, ***P ≤ 0.001). Statistical significance was analyzed using two-way ANOVA with *P* values adjusted using Holm–Sidak correction method.

To measure the diffusion of membrane receptors using single particle tracking, time-lapse movies were acquired using TIRF microscopy. Figure 1a shows the tracks obtained from a series of 300 frames capturing TLR2 diffusion on the basal membrane of a RAW 264.7 cell (Supplementary Movie 1). SPT (*cf.* Methods) was used to generate the tracks for each individual particle. First, the diffusion coefficient of each track was obtained from the mean-squared displacement data and the fit to Eq. (1) (only tracks with ≥ 10 steps were analyzed). The diffusion coefficients (mean ± SD) calculated from this analysis for TLR2 and CD14 in 15 RAW 264.7 cells were *D*_TLR2-Basal_ = 0.08 ± 0.02 μm^2^/s and *D*_CD14-Basal_ = 0.13 ± 0.02 μm^2^/s, respectively. A comparison of the diffusion coefficients versus track length did not reveal any correlation (Supplementary Fig. S1). This verified that the diffusion measurements were not skewed by the number of steps recorded for each particle. For a global diffusion analysis, we created a combined track by concatenating the individual tracks in random order (Fig. 1b), which did not reveal any overall drift or directed motion. The diffusion coefficient was then calculated by fitting Eq. (1) to the MSD data for lag time *τ* ≤ 0.5 s (the mean single-track length for the data set), as shown in Fig. 1c. The average diffusion coefficients calculated using this approach were *D*_TLR2-Basal_ = 0.07 ± 0.02 μm^2^/s and *D*_CD14-Basal_ = 0.11 ± 0.02 μm^2^/s for TLR2 and CD14, respectively. As shown in Fig. 2, both MSD measurements (i.e., individual and combined tracks) reveal that the mean diffusion coefficient of CD14 is higher than TLR2 in the basal membrane. Similarly, fitting Eq. (2) to the step-size distribution data (Fig. 1d) yielded average diffusion coefficients of *D*_TLR2-Basal_ = 0.07 ± 0.01 μm^2^/s and *D*_CD14-Basal_ = 0.11 ± 0.02 μm^2^/s for TLR2 and CD14, respectively, measured from the first lag time *τ* = 0.06 s. In addition, the immobile fraction was different between receptors (20% in TLR2 vs. 15% in CD14).

### Raster image correlation spectroscopy

**Figure 3:**
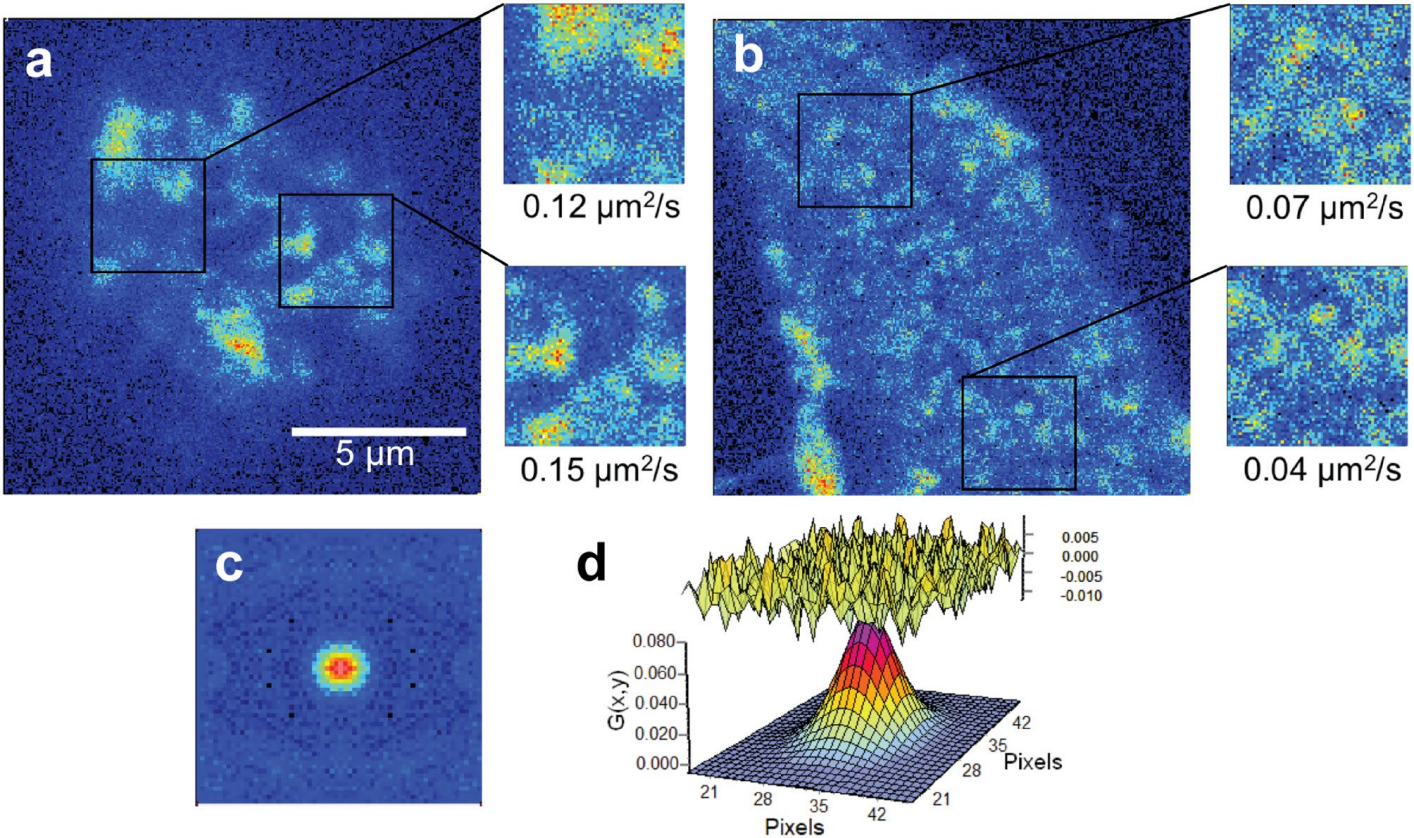
Diffusion measurement of TLR2 using RICS on a macrophage plasma membrane. (**a**) Apical and (**b**) basal membranes of RAW 264.7 macrophage expressing TLR2 receptor; two 64 × 64 frames (3.2 × 3.2 μm^2^) in each membrane show the regions of interest (ROI) and the diffusion coefficient calculated corresponding to each region. (**c**) 2D representation of RICS autocorrelation function for diffusion of 0.07 μm^2^/s. (**d**) Plot of the residues (upper surface) and the 3D representation of the fit to the function (lower surface) for the plot shown in (**c**).

We also measured the lateral diffusion of TLR2 and CD14 in the plasma membrane of RAW 264.7 macrophages using raster image correlation spectroscopy. Receptor diffusion was measured in the apical and basal membranes for comparison (Fig. 3a,b). The basal membrane was defined as the bottom membrane plane, where the cell was in contact with glass, whereas the apical membrane was defined by focusing on the highest observable plane through the cell. A minimum of three measurements in the regions of interest (ROI), in each of the two membrane sections, was acquired for 15 individual cells to determine the diffusion coefficient. The value of the diffusion coefficient was obtained from fitting to the autocorrelation function (Fig. 3c,d). Similar RICS analysis was performed to measure CD14 diffusion.

**Figure 4:**
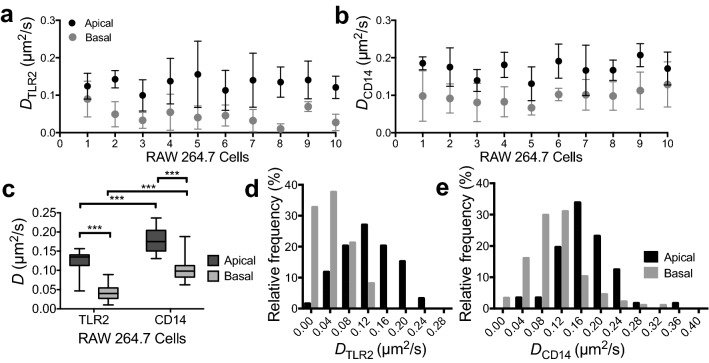
Diffusion coefficients of TLR2 and CD14 in the plasma membranes of RAW 264.7 macrophages obtained through raster image correlation spectroscopy. Diffusion *D* of (**a**) TLR2 and (**b**) CD14 in the apical and basal membranes measured from a minimum of three separate ROIs in each membrane section; mean ± SD for a sample of 10 individual cells is presented. (**c**) Boxes show 25th–75th percentiles with whiskers extending to minimum and maximum values measured from 15 cells. Statistical analysis was performed using paired *t*-test for comparison of apical and basal diffusion, and independent *t*-test between the two receptors (***P ≤ 0.001). Kolmogorov–Smirnov test was performed to check the validity of the normality assumption for the paired *t*-test and Levene’s test of homogeneity of variances was used for the independent *t*-test (Supplementary Tables S1–S2). Relative frequency of the diffusion coefficients measured for (**d**) TLR2 and (**e**) CD14 from a total of 15 cells for each receptor; bin centers shown in the x-axis. Data is from 15 cells with at least 3 independent ROI measurements in each of the apical and basal membranes.

Both TLR2 and CD14 receptors were found to have faster diffusion in the apical membrane (*D*_TLR2-Apical_ = 0.12 ± 0.03 μm^2^/s; *D*_CD14-Apical_ = 0.18 ± 0.03 μm^2^/s) than in the basal membrane (*D*_TLR2-Basal_ = 0.04 ± 0.02 μm^2^/s; *D*_CD14-Basal_ = 0.10 ± 0.03 μm^2^/s), as shown in Fig. 4a,b. The paired comparison *t*-test (Fig. 4c) confirmed the statistical significance of this difference for both CD14 (*P* ≤ 0.001) and TLR2 (*P* ≤ 0.001). Moreover, CD14 diffusion was faster than TLR2 (*P* ≤ 0.001) when similar membrane sections were compared for mobility of these receptors. Figure 4d,e demonstrates the relative frequencies of diffusion coefficients for TLR2 and CD14 in the apical and basal membranes. Approximately 33% of measured ROIs for TLR2 and ~ 3.5% for CD14 had very slow diffusion (*D* ≤ 0.02 μm^2^/s) in the basal membrane. In contrast, similar range of diffusion coefficients was found in only ~ 1.7% and 0% of the ROIs analyzed in the apical membranes for TLR2 and CD14, respectively.

## Discussion

Among the broad repertoire of receptors on the plasma membrane of macrophages, TLRs and their coreceptors play significant roles in recognition of invading pathogens and initiation of inflammatory responses. TLR2 and CD14 are specifically involved in the detection of bacterial lipopeptides and lipoproteins^28^. The important role of TLR2 and CD14 is evident in the increased susceptibility to infections as a result of dysregulation of these receptors^29^. Consequently, diffusion measurements of these membrane receptors can provide fundamental insights into their behavior and uncover the underlying mechanisms leading to changes in TLR signaling.

Four major families of fluorescence-based microscopy techniques (i.e., FRAP, FCS, ICS, and SPT) along with their extended modalities provide a versatile toolbox for analyzing the dynamics of membrane components. Each technique has their specific advantages, and the choice of method depends on the particular application. A number of comparative studies have been conducted to determine the accuracy and differences in diffusion measurements obtained from FCS and FRAP^30^, FCS and SPT^31^, FCS and RICS^32^. The results of these comparative studies can help with the selection of appropriate techniques for specific measurements on the membranes. The aim of this study was to compare the diffusion coefficients for two types of plasma membrane receptors in macrophages, TLR2 and CD14, using two different fluorescence-based imaging techniques. To our knowledge, this is the first study that has directly compared diffusion measurements from SPT and RICS.

Our experimental data showed similar results using SPT and RICS for the diffusion coefficients of CD14 in the basal membranes (Table 1). TLR2 diffusion obtained from RICS in the basal membrane was slower compared with SPT results, which could be associated with the ensemble nature of RICS and its sensitivity to the presence of a large number of particles with slow diffusion that can yield a dominant correlation pattern. The apical membrane cannot be analyzed using TIRF microscopy, as TIRF-SPT is limited to monitoring species close to the glass surface; therefore, only the basal membranes could be directly compared. To date, there are no previous reports of TLR2 diffusion in RAW 264.7, the most commonly used macrophage cell line. The diffusion coefficients we measured using RICS and SPT in the basal membrane are lower than the reported value of 0.17 ± 0.03 μm^2^/s measured by FRAP in Chinese hamster ovary cells (CHO) transfected with TLR2^24^. Given that the size of FRAP observation region can influence the measurement of the fractional population that diffuses slow or fast, it is possible that the slow population in FRAP was not detected. It is also worth noting that receptor expression in these two cell lines might be significantly different, which could also contribute to the differences observed. Whether this inconsistency is due to different types of cells, or the fluorescence imaging technique utilized is not clear and could be the subject of further comparative studies. However, similar to FRAP measurements, our MSD data shows simple Brownian diffusion for unstimulated TLR2. Our results are also in agreement with previous SPT measurements of CD14 on RAW 264.7. The diffusion coefficient we obtained from SPT and RICS (Table 1) is comparable to the reported value 0.14 ± 0.02 μm^2^/s, with MSD plot showing no deviation from Brownian motion^33^. This data further confirms the compatibility of RICS with SPT, which is widely applied in diffusion measurements.

**Table 1:** Diffusion coefficients of TLR2 and CD14 in the plasma membrane of RAW 264.7 measured by RICS and SPT.

Technique	Analysis	Membrane section	Receptor	*D *(Mean ± SD) μm^2^/s		
RICS		Apical	TLR2	0.12 ± 0.03		
		Basal	TLR2	0.04 ± 0.02		
		Apical	CD14	0.18 ± 0.03		
		Basal	CD14	0.10 ± 0.03		
				**All tracks**	**Immobile removed**	**Number of tracks**^**d**^** (immobile)**
SPT	i-MSD^a^	Basal	TLR2	0.08 ± 0.02	–	–
	c-MSD^b^	Basal	TLR2	0.07 ± 0.02	0.08 ± 0.02	144 (29)
	SSD^c^	Basal	TLR2	0.07 ± 0.01	0.08 ± 0.02	144 (29)
	i-MSD	Basal	CD14	0.13 ± 0.02	–	–
	c-MSD	Basal	CD14	0.11 ± 0.02	0.13 ± 0.02	244 (37)
	SSD	Basal	CD14	0.11 ± 0.02	0.12 ± 0.02	244 (37)

^a^i-MSD = MSD of individual tracks, ^b^c-MSD = MSD of combined tracks, ^c^SSD = Step-size distribution, ^d^Number of analyzed tracks ≥ 10 steps.

The GPI-anchored receptor CD14 had faster diffusion than the transmembrane receptor TLR2 in RAW 264.7 macrophages. This finding is in agreement with reported data on several chimeras of the two groups of membrane proteins (i.e., transmembrane proteins that were converted to GPI-linked proteins, and the GPI-linked proteins that were transformed to membrane-spanning proteins)^34^. Early FRAP experiments performed on chimeric proteins, including the GPI-linked Thy-1, human placental alkaline phosphatase, and murine surface antigen Ly6E, resulted in diffusion coefficients **∼**2 to 5 fold faster than transmembrane proteins such as vesicular stomatitis virus G (VSV-G)^34^. However, not all GPI-linked proteins exhibit fast lateral mobility, such as PH-20, a GPI-linked surface antigen with highly restricted diffusion on testicular sperm before maturation^35^. The difference we observed in the diffusion coefficients of TLR2 and CD14 may be associated with their mechanism of signaling. CD14 is a GPI-linked receptor (i.e., lacks a cytoplasmic domain), which likely enables faster lateral mobility than the transmembrane TLR2. Whether this contributes to its ability to rapidly bind and deliver microbial ligands to slower moving TLRs is not known. Alternatively, whether CD14 diffuses faster than TLR2 to quickly occupy the transmembrane receptor-depleted regions or to compensate for the slow arrival of antigen-bound TLRs would require further investigation of the kinetics of receptor-ligand binding events.

Most diffusion measurements reported in the literature are from one membrane section; the basal membrane in SPT and only one of the apical or basal membranes in FRAP experiments, depending on the imaging apparatus. SPT studies are typically performed using TIRF and therefore the reported diffusion coefficients are based on data acquired from the basal membrane in contact with glass. While these measurements can provide valuable insight into the dynamics of certain plasma membrane proteins that mostly reside in the basal membrane, such as those involved in adhesion to substrates or the extracellular matrix, many others have to be analyzed on the apical or lateral membrane and away from the cell-glass interface to capture their true dynamic behavior. Therefore, in recent years new techniques have been developed to image plasma membrane proteins in the sections of the cell membrane where there is no direct contact with the coverslip^36^. For example, single molecule light-sheet microscopy has been used to compare the diffusion of TCR and CD45 receptors on the apical and basal membranes of Jurkat T cells on various surface coatings, which has revealed the impact of cell-surface interactions on the measured diffusion^37^. In contrast, a recent study on 293 T cells using single particle tracking and 3D lattice light-sheet microscopy in combination with direct stochastic optical reconstruction microscopy showed that the mobility of CD56, CD2, and CD45 receptors was unaffected at the cell-coverslip interface, although lower localization of the receptors was detected in the basal membrane^38^. The implication of these studies is that the mobility of certain membrane receptors can be more influenced by cell adhesion to glass coverslip. For macrophages, this is particularly crucial when studying the diffusion of receptors that are involved both in cell adhesion and phagocytosis (i.e., play dual roles), such as integrins^39^ and scavenger receptors^40^.

Diffusion measurements of key immunoreceptors such as TLR2 and CD14 can elucidate their binding and signaling mechanisms, which can in turn lead to rational development of potential therapeutic strategies to tune or amplify their response in cells that are impaired due to aging or disease.

## Conclusion

In summary, we have obtained comparable diffusion measurements using RICS and SPT for the lateral movement of a GPI-anchored receptor and a transmembrane receptor in the plasma membrane of RAW 264.7 macrophages. Both techniques revealed higher diffusion of CD14 compared with TLR2. While RICS allows measuring the diffusion in different planes through the cell body, SPT shows the trajectories of individual particles in the nearest planar section of the membrane in contact with glass. Consequently, RICS and SPT can be used as complementary methods for studying membrane dynamics.

## Methods

### Cell culture

Macrophage cell line RAW 264.7^41^ from American Type Culture Collection (ATCC) was cultured using RPMI-1640 supplemented with 10% fetal bovine serum (FBS), 1% L-glutamine, and 1% penicillin/streptomycin. Twenty-four hours before imaging, the cells were incubated with Trypsin/EDTA (Gibco) for 5 min, then gently lifted using a cell lifter and plated at a density of 100,000 per Glass Bottom dish (35 mm dish, 20 mm Microwell, No. 1.5 coverglass, 0.16–0.19 mm thickness, MatTek, Ashland, MA). Fluorescent microspheres (FluoSpheres, carboxylate-modified, 0.17 μm, excitation: 505 nm; emission: 515 nm, Life Technologies, Invitrogen Molecular Probes, Eugene, OR) were mixed in 50% isopropanol in water and added to the imaging dishes at a density that guaranteed 1–3 microspheres per field of view to serve as the fiducial markers for drift correction. Prior to seeding the cells, the microspheres were fused to the coverglass by heating the dish containing microspheres in solution at 65 °C for 10 min.

### Fluorescence staining

All imaging was performed in RPMI-1640 medium without phenol red (Life Technologies- Gibco). TLR2 and CD14 were visualized in separate experiments using mouse anti-TLR2/CD282 antibody labeled with Alexa Fluor 647 (0.2 mg/mL, BD Biosciences) and mouse anti-CD14 antibody labeled with APC (excitation: 633–647 nm, emission: 660 nm, 0.2 mg/mL, eBioscience), at a 1:1000 dilution in imaging media. Antibody staining was performed at 4 °C for 1 h to prevent receptor internalization, after which the samples were washed with PBS 3 × and the media was replaced with RPMI including 5 mM ascorbic acid to minimize photobleaching during imaging.

### Single particle tracking (SPT)

#### Total internal reflection fluorescence microscopy (TIRF)

SPT imaging was performed on an objective-based TIRF setup built on a Leica inverted microscope stand (Leica DMI6000 B, Germany) and outfitted with an oil-immersion objective of 100×/1.47NA (HCX PL APO, Leica Germany CORR TIRF). The excitation light source was a LMM5 solid state laser launch (Spectral Applied Research Inc., Richmond Hill, ON, Canada) with 488 and 647 nm emission lines. Single particle images were captured by an Andor iXon Ultra EMCCD camera (Andor Technology Ltd., Belfast, U.K). Time-lapse movies of 300 frames were acquired using Micro-Manager software (MMStudio Version 1.4.22) at a frame rate of **∼**16.7 fps (acquisition time of τ_a_ = 60 ms per frame), with a field of view of 512 × 512 pixels and pixel sizes of 97 nm × 97 nm. Live cell imaging was performed at 37 °C using an objective heater (FCS2, Bioptechs Inc., Butler, PA).

#### Particle detection and tracking

The series of frames captured for each cell were analyzed using an in-house program written in Python, under the use of the packages numpy, scipy, pandas, tkinter, and scikit-images, using a single-particle detection and tracking algorithm^42–44^. Each raw image was first processed with a Gaussian and a top-hat filter. The local maxima, i.e., approximate particle positions, were determined and a threshold was applied to generate a binary map of the estimated positions for each particle, which were used as starting values in the particle fitting algorithm. All particles within the raw images were then fitted to a 2D Gaussian model function via least squares optimization. The width and eccentricity of the detections were analyzed from the fitted functions and were excluded if they exceeded predefined thresholds (4 standard deviations of the background). The fitting algorithm determined the particle (fluorescent antibodies) positions with an accuracy of **∼**20 nm^43^. Individual positions in consecutive frames were then linked to generate tracks for each particle using an appropriate search radius determined from simulations (Supplementary Fig. S2–S6). Every link was associated with a cost, representing the travelled distance of the particle in a frame interval. The tracks of individual particles were generated by minimizing the global cost for all links.

To obtain the number of immobile particles, the step-size distribution of the shortest lag time was used from which the standard deviation was extracted. A threshold of three standard deviations (3σ) was used to determine the immobile fraction (stationary particle if end-to-end tracks < threshold).

#### Measuring diffusion coefficient

Diffusion coefficients were calculated using three different methods from the generated tracks. We first used each individual track to calculate the mean-squared displacement (MSD) and the diffusion coefficient *D* from:

1 $$\left\langle {\left( {\Delta r} \right)^{2} } \right\rangle \left( \tau \right) = 4D\tau .$$

In the second approach, all tracks generated within a single cell were concatenated to create a master trajectory and the combined-MSD (c-MSD) was used to determine *D* from Eq. (1).

In the final approach, the distribution of displacements was analyzed using Eq. (2) for all tracks. For a simple diffusion process, the probability density function for a particle in one dimension is Gaussian and its widths is time dependent according to:

2 $$P\left( {\Delta x, \tau } \right) = \frac{1}{{\sqrt {4\pi D\tau } }}\exp \left[ { - \frac{{\Delta x^{2} }}{4D\tau }} \right],$$

where Δ*x* is the displacement after a lag time *τ*. The diffusion coefficients were obtained for different lag times, by fitting to the distributions of displacements.

### Raster image correlation spectroscopy (RICS)

#### Confocal imaging

Raster-scan images were collected using confocal laser-scanning microscopy (CLSM, Olympus FluoView FV1000, Central Valley, PA) on an inverted microscope stand outfitted with a 60×/1.20NA UPLSAPO water-immersion objective. Excitation was from 647-nm laser line attenuated to 0.5–1.0% nominal power. For scanning the basal membrane, the plane of focus was set as close as possible to the bottom membrane plane, where the cell was making contact with glass. The apical membrane was found by focusing on the highest observable plane through the cell.

For raster image correlation spectroscopy (RICS), 256 × 256 pixel images were collected using FluoView imaging software (Olympus FluoView 1.7a) at 16.4 × zoom, corresponding to a pixel size of 50 nm. For raster-scanning live cells, the pixel dwell time was set to 40 μs/pixel (line time 11.264 ms). Stacks of 50 images were captured with no delay between frames. Imaging of live cells was performed at 37 °C using a stage-top incubator (Tokai Hit, Shizuoka, Japan).

#### RICS diffusion measurements

Diffusion data was obtained using the SimFCS software^12^ (The Laboratory for Fluorescence Dynamics, University of California, Irvine; available at www.lfd.uci.edu). Regions of interest (ROI) were selected with sizes of 64 × 64 pixels (3.2 × 3.2 μm^2^) within each cell after analyzing the autocorrelation functions obtained from different ROI sizes (Supplementary Fig. S7–S8). The diffusion coefficient was measured using the known parameters of pixel dwell time, line time, retracing time, size of each pixel, and the beam waist. A focal volume waist (ω_0_) of 0.24 μm was measured according to the previously established methods^12^. For each stack of images, the RICS function was calculated as the average of all images of the stack. Data was obtained from 15 cells for each receptor, using a minimum of three separate ROIs measured in each of the apical and basal membranes.

#### Principles of RICS

The principles of RICS are explained in detail in the seminal papers by Gratton and Digman^10,11^. Here we briefly reiterate the autocorrelation functions for reference purposes. The scanning function that relates time with space, i.e., the spatio-temporal correlation is defined as:

3 $$\tau \left( {\xi ,\psi } \right) = \tau_{p} \xi + \tau_{l} \psi ,$$

where *τ*_*p*_ and *τ*_*l*_ denote pixel dwell time and line scan time, respectively. *ξ* and *ψ* are the spatial displacements (in pixels) in the horizontal and vertical direction of scan in the raster image, respectively. The normalized spatial correlation function of the pixel fluorescence intensity fluctuations is defined as:

4 $$G_{s} \left( {\xi ,\psi } \right) = \frac{{\left\langle {\delta I\left( {x,y} \right)\delta I\left( {x + \xi ,y + \psi } \right)} \right\rangle_{x,y} }}{{\left\langle {I\left( {x,y} \right)} \right\rangle_{x,y}^{2} }} = G\left( {\xi ,\psi } \right)S\left( {\xi ,\psi } \right),$$

where *I* (*x*, *y*) is the detected fluorescence intensity at each pixel and δ*I*(*x*, *y*) = *I*(*x*, *y*) − < *I*(*x*, *y*) >_x,y_ are the fluorescene intensity fluctuations around the mean intensity. The autocorrelation function for 3D diffusion is:

5 $$G\left( {\xi ,\psi } \right) = \frac{\gamma }{N} \left( {1 + \frac{{4D\left( {\tau_{p} \xi + \tau_{l} \psi } \right)}}{{\omega_{0}^{2} }}} \right)^{ - 1} \left( {1 + \frac{{4D\left( {\tau_{p} \xi + \tau_{l} \psi } \right)}}{{\omega_{z}^{2} }}} \right)^{ - 1/2} ,$$

where *D* is the diffusion coefficient, *N* is the average number of molecules in the observation volume, and ω_0_ and ω_z_ are the lateral and axial waists of the laser beam at the point of focus. The γ factor accounts for the non-uniform illumination of the excitation volume and is equal to 0.3535 for 3D and 0.5 for 2D Gaussian point spread functions (PSF)^45^. For 2D diffusion, the last factor in Eq. (5) is omitted.

*G* (*ξ*, *ψ*) is the autocorrelation function that results from molecular diffusion only. Since the PSF spans over several pixels in the imaging plane, the correlation for the contribution of the scan itself should be also taken into account. For square pixels with dimension δr × δr the correlation for the scan is given by:

6 $$S\left( {\xi ,\psi } \right) = \exp \left( {\frac{{\left[ {\left( {\frac{\xi \delta r}{{\omega_{0} }}} \right)^{2} + \left( {\frac{\psi \delta r}{{\omega_{0} }}} \right)^{2} } \right]}}{{1 + \frac{{4D\left( {\tau_{p} \xi + \tau_{l} \psi } \right)}}{{\omega_{0}^{2} }}}}} \right).$$

### Statistical analysis

Statistical analysis and plotting were performed using SPSS software (IBM SPSS Statistics 21) and Prism 7.0a (GraphPad Software Inc.), respectively. Differences were considered statistically significant at *P* values of < 0.05.

## Supplementary information

Supplementary Information

Supplementary Information
