# Supplementary material for: Lateral diffusion of CD14 and TLR2 in macrophage plasma membrane assessed by raster image correlation spectroscopy and single particle tracking
Source: Sci Rep. 2020 Nov 9;10:19375. doi: 10.1038/s41598-020-76272-2 (PMC7652837; doi:10.1038/s41598-020-76272-2)
Supplement: Supplementary file 2 — Supplementary Information [file 41598_2020_76272_MOESM2_ESM.docx]

## Supplementary Information

Lateral Diffusion of CD14 and TLR2 in Macrophage Plasma Membrane Assessed by Raster Image Correlation Spectroscopy and Single-Particle Tracking

Sara Makaremi^1^, Markus Rose^2^, Suman Ranjit^3^, Michelle A. Digman^4^,

Dawn M.E. Bowdish^5,6^*, Jose M. Moran-Mirabal^1,7^*

^1^School of Biomedical Engineering, McMaster University, ON, Canada

^2^Department of Physics & Astronomy, McMaster University, ON Canada

^3^Department of Biochemistry, Molecular & Cellular Biology, Georgetown University, Washington DC, USA

^4^Department of Biomedical Engineering, University of California Irvine,

Irvine, CA, USA

^5^Department of Pathology and Molecular Medicine, McMaster University, ON, Canada

^6^MG DeGroote Institute for Infectious Diseases, McMaster University, ON, Canada

^7^Department of Chemistry and Chemical Biology, McMaster University, ON Canada

email for correspondence: [mirabj@mcmaster.ca](mailto:mirabj@mcmaster.ca), [bowdish@mcmaster.ca](mailto:bowdish@mcmaster.ca)

**Supplementary Movie 1.**

Time-lapse movie of a RAW 264.7 macrophage cell imaged under Total Internal Reflection Fluorescence Microscopy. Red channel: TLR2 visualized in the basal plasma membrane using mouse anti-TLR2/CD282 antibody labeled with Alexa Fluor 647. Green channel: Fluorescent microspheres fused to the glass to serve as the fiducial markers for drift correction. Frame rate: 16.7 fps (acquisition time of τa = 60 ms per frame), and pixel size: 97 nm × 97 nm. Single particle tracking results for the same cell are shown in the paper (Figure 1).


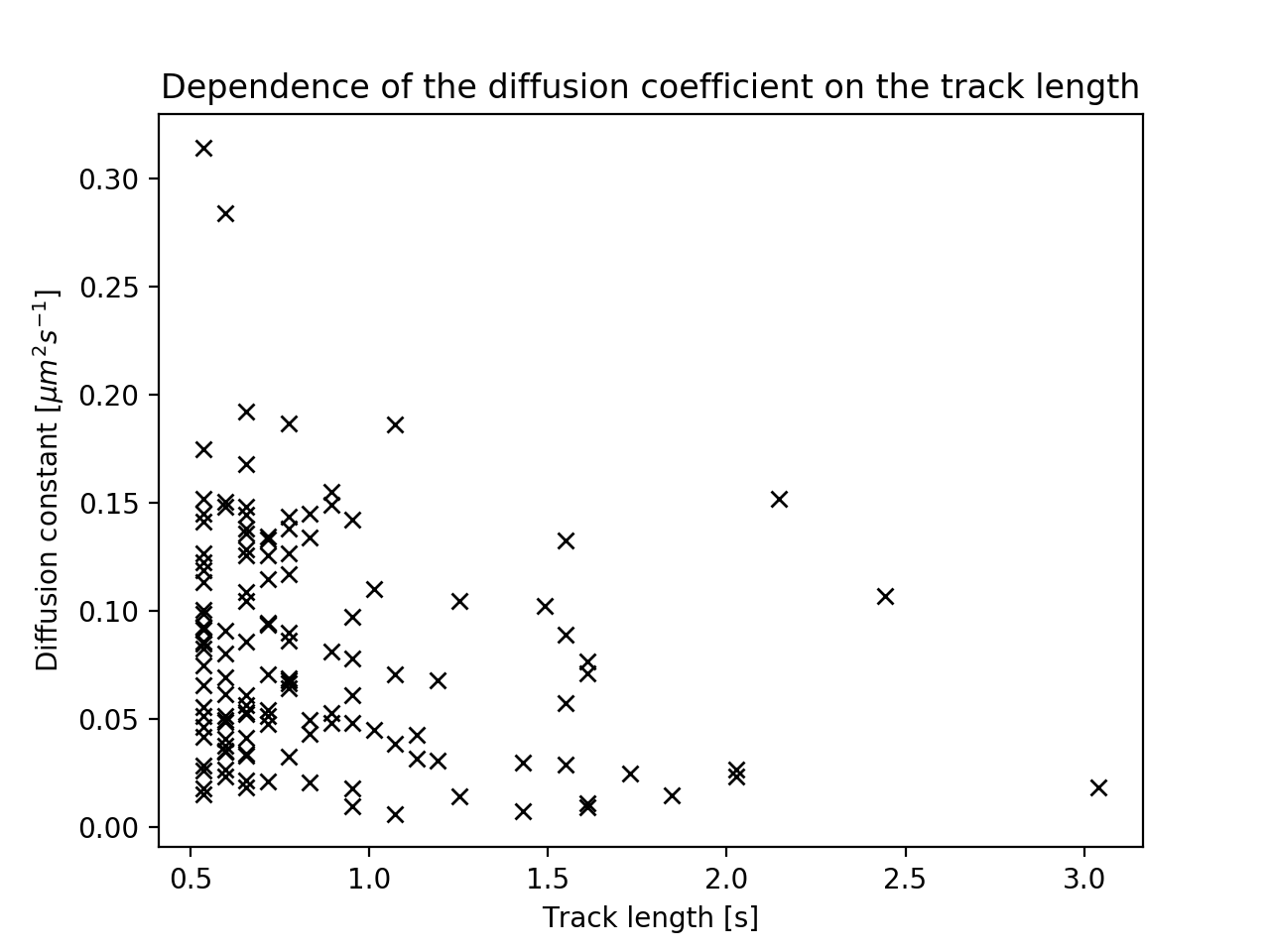


**Figure S1.** Measured diffusion is independent of track length. The diffusion coefficients and track lengths for individual tracks obtained from tracking TLR2 in the plasma membrane of RAW 264.7 cell (Figure 1A) do not show any correlation.

**Table S1.** SPSS output for Kolmogorov-Smirnov test to check the validity of normal distribution assumption for the paired *t*-test. There is no statistically significant deviation from normality (*P* > 0.05) for TLR2 and CD14 diffusion data in the apical and basal membranes of 15 macrophage cells.


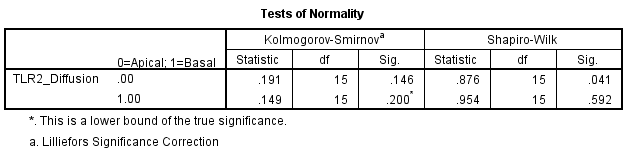


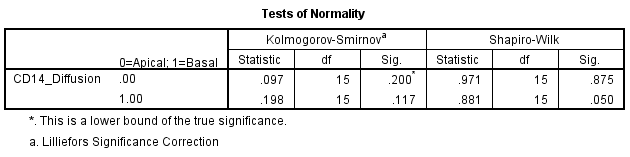


**Table S2.** SPSS output for Levene’s test of homogeneity of variances to check the validity of equal variances assumption for the independent *t*-test. There is no statistically significant difference (*P* > 0.05) between variances of the diffusion data in the apical and basal membranes of 15 macrophage cells.


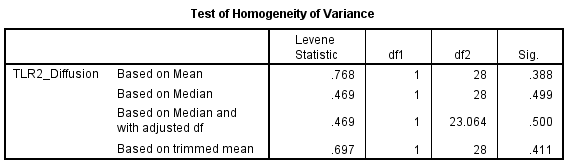


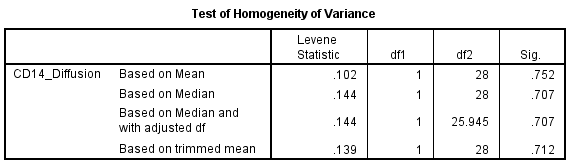


**Search Radius Optimization for a Fixed Diffusion Coefficient**

When tracking a single particle in time, the algorithm will search for a particle within a similar location in subsequent frames. The location is defined as a circle of radius r_SR_ (Search Radius) centered on the previous particle localization. The choice of r_SR_ is crucial for an unbiased analysis. Confining the search area can be beneficial for analysis time, but can lead to premature track termination and lower measured diffusion coefficient values. For unreasonably large r_SR_ a particle that has terminated, *e.g*. due to photobleaching, might falsely be linked to another particle and bias the measured diffusion coefficient to larger values. Optimizing r_SR_ is therefore a requirement for valid diffusion coefficient measurement.

To determine the optimal search radius (SR) for track continuation in subsequent frames, different imaging and linking conditions were simulated and compared to the expected value of a restricted Gaussian step size distribution. For both simulation and theoretical considerations two diffusion coefficients of *D* = 0.1 μm^2^ s^-1^ and *D* = 0.15 μm^2^ s^-1^ was assumed. The pixel size of 0.1 μm and frame interval of τ = 60 ms was kept constant.

To arrive at the expected value, 10^6^ displacements (*x*i, *y*i) were sampled from a Gaussian distribution with zero mean and a variance of 2Dτ and restricted by a search radius $r_{SR}\geq\sqrt{x_{i}^{2}+y_{j}^{2}}$.

The accepted displacements were combined into a master track and the diffusion coefficient was obtained via a linear mean-squared displacement fit.

To see the dependence of the measured diffusion coefficient on the search radius, simulations for particle concentrations of 0.031 μm^-2^, 0.092 μm^-2^ and 0.153 μm^-2^ were created with continuous tracks, as well as emission state switching tracks to emulate blinking. Continuous tracks were created for negligible noise (*S/N* > 700), as well as a signal-to-noise ratio of 1.7. When blinking was active, the switching probabilities were chosen to be *p*on = 0.3 and *p*off = 0.1. All simulated videos had a length of 1000 frames. The videos were analyzed with the in-house single-particle-tracking algorithm [1] and the search radius was varied from 0.5 px to 10 px.

**Theory**

Limiting the linking process with r_SR_ is in effect removing the tail ends of the Gaussian step size distribution for each displacement coordinate. To recreate this, 10_6_ displacements ⃗*rj* = (∆*xj* , ∆*yj* ) are sampled component-wise from a 1D Gaussian distribution of shape

$\Theta\left[ \Delta x_{j} \right]=exp\left\{ -\frac{\Delta x_{j}^{2}}{4D\tau} \right\}$ . (1)

The introduction of r_SR_represents a restriction of the displacements of ${r_{SR}}^{2}\geq{⃗r}_{k}^{2}$. Displacements that follow this restriction are then combined into a master track by append ing displacements ${}_{r}^{\to}{(t|r_{SR})}=\sum_{k=1}^{t<k.\tau} {⃗r}_{k}$. The resulting diffusion coefficient can be obtained from a linear MSD fit to the master track data

$\left\langle{\vec{\Delta}r}^{2}(t) \right\rangle=\left\langle{(⃗r(k\tau+t)-⃗r(k\tau))}^{2} \right\rangle_{k}=4D_{SR}t$ . (2)

**Simulation**

Videos were simulated by creating particle tracks for *N* particles with *K* steps for the diffusion coefficient *D* and step interval *τ*. The particle position in frame k of the video is given by

$\vec{r}_{n}\left( k.\tau\right)=\vec{r}_{n}\left( \left( k-1 \right).\tau\right)+ \Delta⃗r(D,\tau)$ , (3)

with components of $\Delta⃗r$ taken from (1).

Each image was created by adding the intensities of all visible *N* particles for each pixel.

$I\left( \vec{r}_{px} \right)=\sum_{n=1}^{N} I_{G}\left( {\vec{r}_{n}-\vec{r}}_{px} \right)+B_{Gauss}(\vec{r}_{px})$ , (4)

with the diffraction limited spot intensity

$I_{G}(\vec{\Delta}r)=exp\left\{ -\frac{{\vec{\Delta}r}^{2}}{2\sigma^{2}} \right\}$ , (5)

and a Gaussian background noise *B*_Gauss_ with a global average background intensity of *B*, a standard deviation σ_Β_ of and a resulting distribution

$\Phi[B_{Gauss}]= \frac{1}{\sqrt{4\pi\sigma_{B}^{2}}}$ $exp\left\{ -\frac{{(B_{Gauss}-B)}^{2}}{2\sigma_{B}^{2}} \right\}$ . (6)

The resulting images *I*(⃗*r*px) were then converted to 16-bit integer values *i_x,y_* and modulated with Poisson noise resulting in the output image *j_x,y_* with distribution

$\Psi\left[ j_{x,y} \right]= e^{-i_{x,y}}\frac{{( i_{x,y})}^{j_{x,y}}}{j_{x,y}!}$ . (7)

The signal-to-noise ratio is determined as the ratio of the squared signal and the background intensity variance. With the definition of the signal being the difference of average particle intensity value and the average background *S* = *I* – *B*

$\frac{S}{N}=\frac{S^{2}}{\sigma_{B}^{2}}= \frac{{(I_{av}-B)}^{2}}{\sigma_{B}^{2}}$ (8)

To reproduce the blinking behavior observed in fluorescence experiments, particles were given the ability to switch ‘on’ and ‘off’ with probabilities *p*_on_ = 0.3 and *p*_off_ = 0.1, respectively. The implementation sees the particles state described as an additional Markov chain of Boolean values *s*_n_ (*t*), with *s*_n_ (*t*)=1 representing the ‘on’ state. This is a simple modification of (4) with the Markov chain

$I\left( \vec{r}_{px},t \right)= \sum_{n=1}^{N} I_{G}(\vec{r}_{n}\left( t \right)-\vec{r}_{px}). s_{n}(t)+B_{Gauss}(\vec{r}_{px}).$ (9)

**Simulation Setup and Considered Conditions**

For all further considerations the pixel size of 0.1 μm and the frame interval of *τ* = 60 ms, as well as the assumed diffusion coefficient *D* = 0.1 μm^2^/s or *D* = 0.15 μm^2^/s, were kept constant. In total, the simulation was carried out for nine situations. Each scenario of a continuous track with negligible noise (*Iav* = 3000, *B* = 300, *σB* = 100), a continuous track with S/N ≈ 1.7 (*Iav* = 3000, *B* = 1000, *σB* = 1500) and a track with blinking and negligible noise was carried out for three particle concentrations of 0.031 μm^-2^ (N = 20 particles per 256 × 256 pixel), 0.092 μm^-2^ (*N* = 60 particles per 256 × 256 pixel), and 0.153 μm^-2^ (*N* = 100 particles per 256 × 256 pixel).

**Detection and Tracking**

The simulated data is detected and tracked using our in-house algorithms (Source: https://github.com/MarkusRose/ParticleTracker). Each data set brings 5 observables: Number of Tracks (Num), Length of tracks (Len), Diffusion coefficient of individual tracks via MSD (Dindiv), Diffusion coefficient of the combined track via MSD (D), and Diffusion coefficient of the combined track through Gaussian step size distribution (Dstep).

**Simulation Results**

The simulation results shown in Figures S2-S6 were obtained using three particle concentrations (20, 60, 100 particles per 256 × 256) with and without blinking, a signal-to-noise ratio of 1.7, and a constant diffusion coefficient of *D* = 0.1 μm^2^ s^-1^.


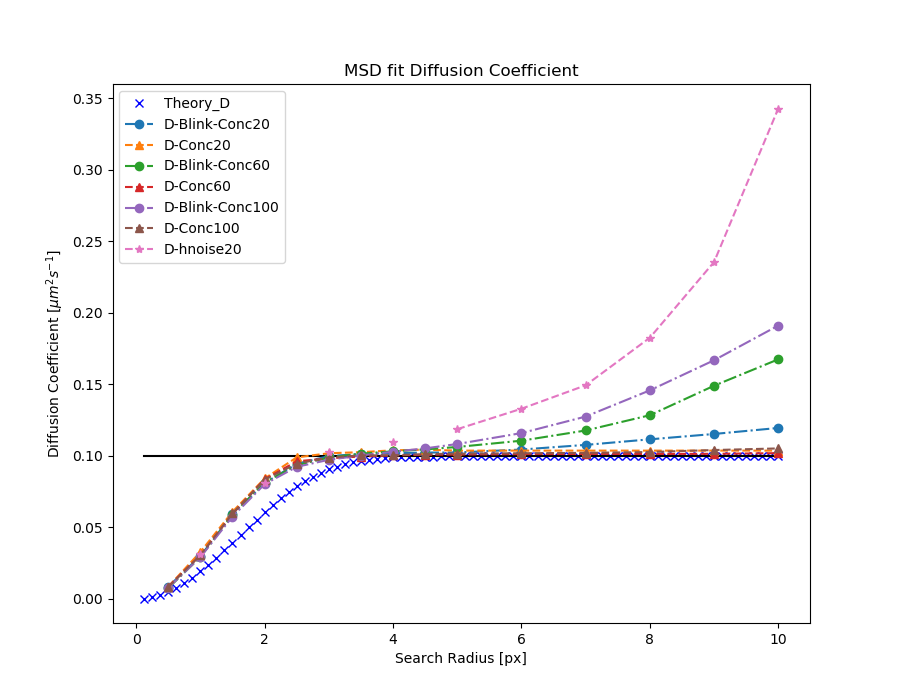


**Figure S2.** Simulation results showing the change in the diffusion coefficient obtained from MSD of combined tracks for a range of search radii.


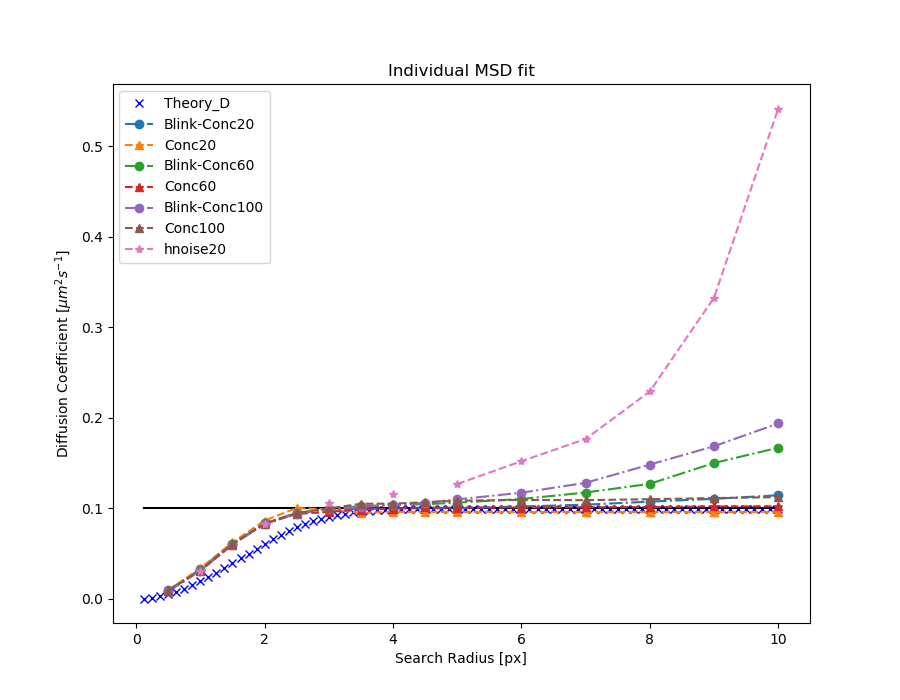


**Figure S3.** Simulation results showing the change in the diffusion coefficient obtained from MSD of individual tracks for a range of search radii.


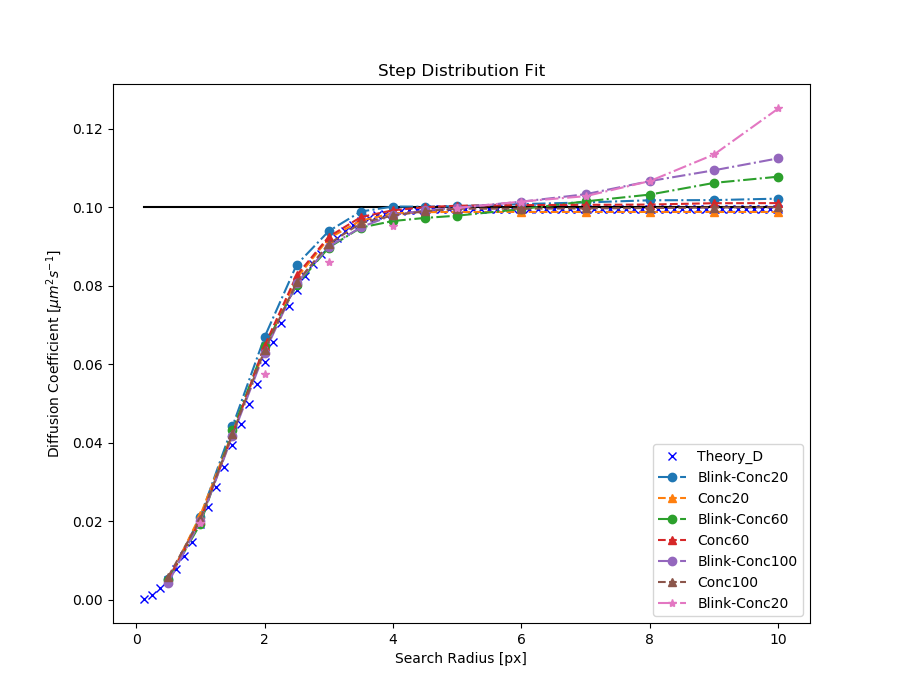


**Figure S4.** Simulation results showing the change in the diffusion coefficient obtained from step-size distribution for a range of search radii.


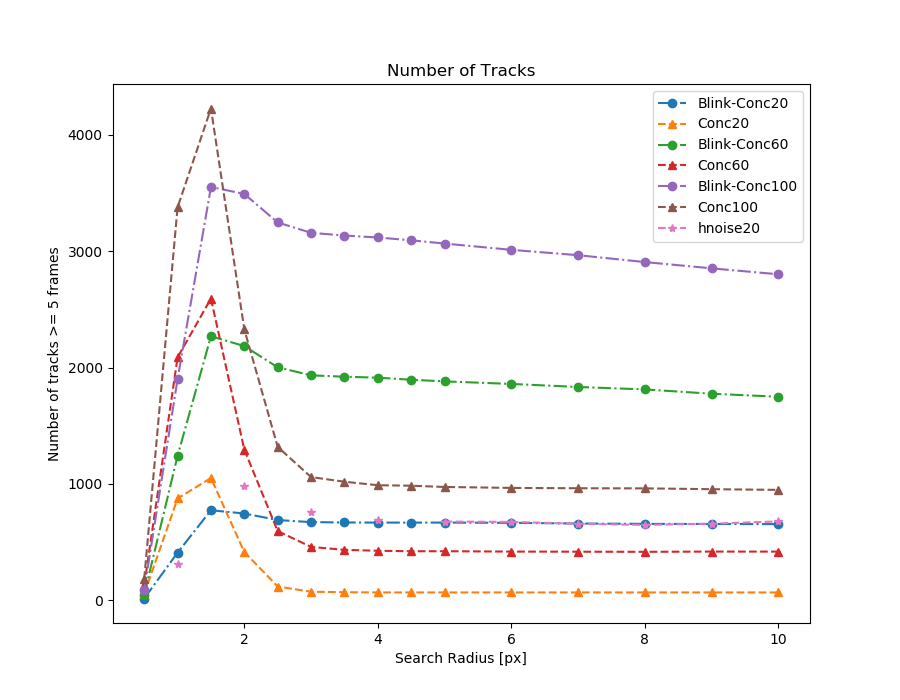


**Figure S5.** Simulation results showing the change in the number of generated tracks for a range of search radii.


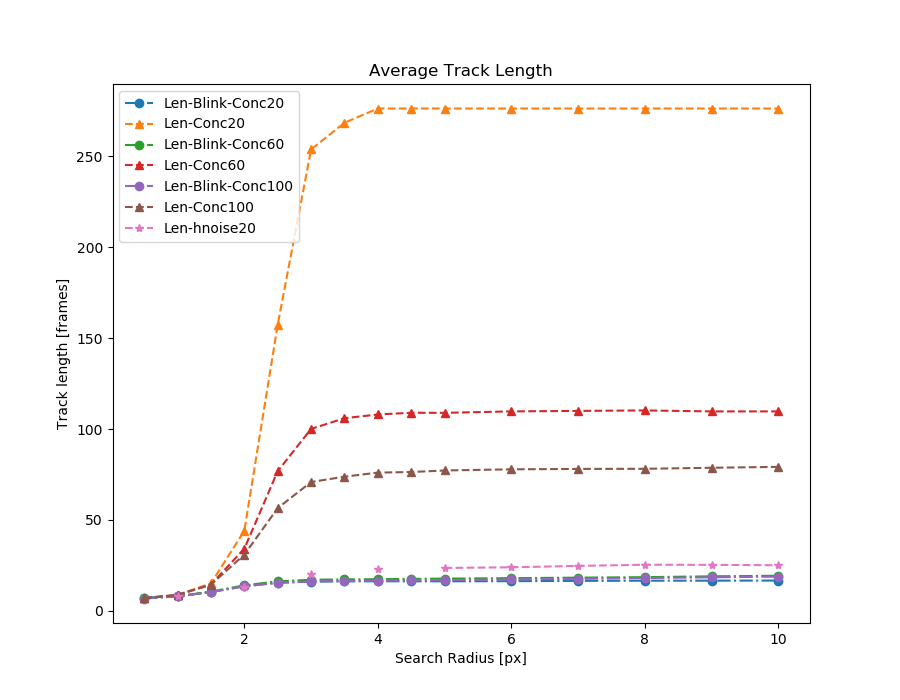


**Figure S6.** Simulation results showing the change in the average track length for a range of search radii.

**Optimization of ROI Size for Raster Image Correlation Spectroscopy**

The effect of the size of region of interest (ROI) on the measured diffusion coefficient has been previously investigated and reported by Brown *et al* [2]. The ROI size affects the diffusion measurement if it cuts off the autocorrelation function (ACF) at longer correlation distances, which can lead to under-estimation of the diffusion coefficient [2]. To verify that our ROI size of 64 × 64 (3.2 × 3.2 μm^2^) did not cut off the ACF, we captured the central H and V line (*i.e*., horizontal and vertical cross sections of the ACF) as well as the fit to the horizontal line only using SimFCS in order to show the pixel shifts for different ROI sizes. Similar trend was observed in the pixel shifts for the ROIs of 64 × 64, 128 × 128, and 256 × 256, while the 32 × 32 (1.6 × 1.6 μm^2^) ROI size did not yield an appropriate fit to the ACF and did not completely level off; therefore, resulted in negative values (Figure S7 and S8). This result is in line with what Brown *et al*. have reported for slightly smaller pixel sizes 32 × 32 (1.5 × 1.5 μm^2^) [2].


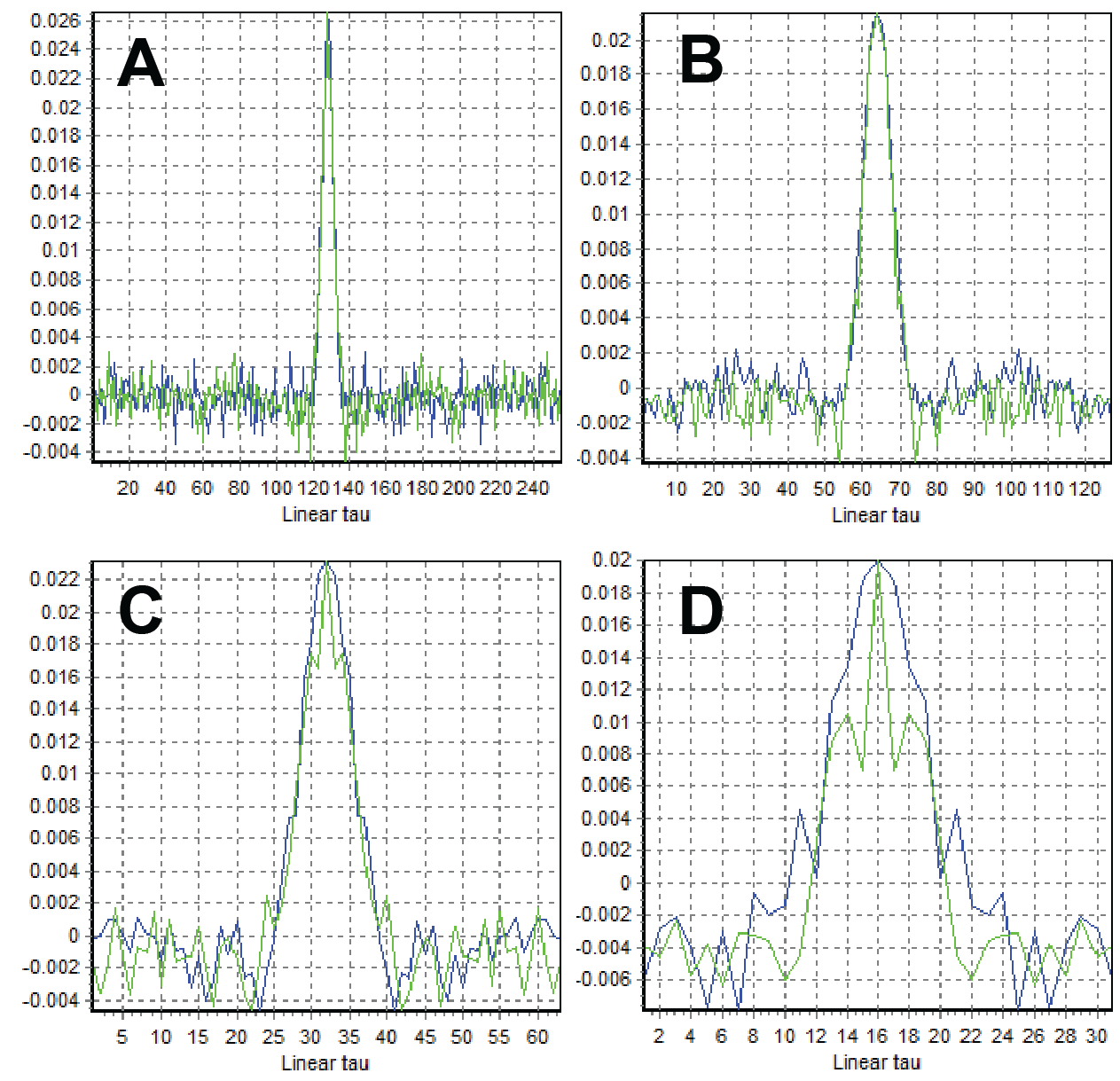


**Figure S7.** Central H and V line (*i.e*., horizontal and vertical cross sections of the ACF) for different ROI sizes obtained from SimFCS: (A) 256 × 256, (B) 128 × 128, (C) 64 × 64, (D) 32 × 32.


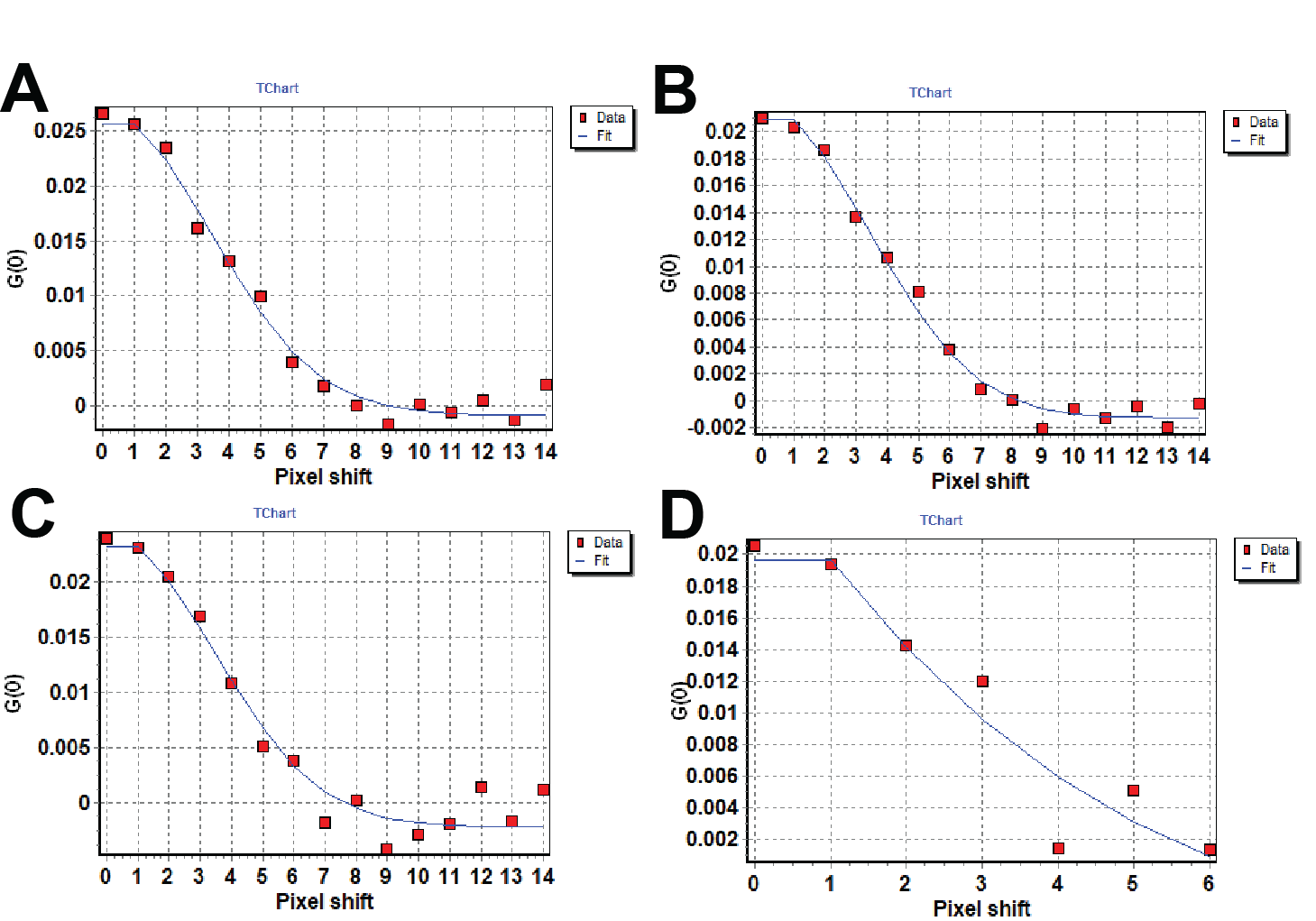


**Figure S8.** Fit to the horizontal ACF and pixel shifts shown for different ROI sizes obtained from SimFCS: (A) 256 × 256, (B) 128 × 128, (C) 64 × 64, (D) 32 × 32.

It should be taken into account that Brown *et al*. measured the diffusion of free EGFP in solution, which has a relatively fast diffusion coefficient (~87 μm^2^/s) compared to the diffusion of EGFP in live cells in the cytosol (~8 to 16 μm^2^/s) [3] and other membrane bound proteins (~0.1 μm^2^/s) [4]. For fast diffusion we can avoid “chopping off” that broadening of the correlation function by choosing a larger ROI size (256 × 256 or 128 × 128). However, if the diffusion is slow like in our measurements with membrane bound proteins, a 64 × 64 ROI will not be a problem because the function will decay without the issue of broadening in space. In other words, with slow diffusion the function does not broaden (*i.e*., the probability is significantly lower to see the slow particles further along the x-axis than when the particles are diffusing fast).

**References:**

[1] <https://github.com/MarkusRose/ParticleTracker>

[2] Brown, C.M. et al. Raster image correlation spectroscopy (RICS) for measuring fast protein dynamics and concentrations with a commercial laser scanning confocal microscope. *J. Microsc.* **229**(0 1), 78-91 (2008).

[3] Ruan, Q., Chen, Y., Gratton, E., Glaser, M., Mantulin, W.W. Cellular characterization of adenylate kinase and its isoform: two-photon excitation fluorescence imaging and fluorescence correlation spectroscopy. *Biophys J*. **83**(6), 3177-87 (2002).

[4] Hegener, O. et al. Dynamics of beta2-adrenergic receptor-ligand complexes on living cells. *Biochemistry* **43**(20), 6190-9 (2004).
